# Integrated Pest Management of Coffee Berry Borer in Hawaii and Puerto Rico: Current Status and Prospects

**DOI:** 10.3390/insects8040123

**Published:** 2017-11-14

**Authors:** Luis F. Aristizábal, Melissa Johnson, Suzanne Shriner, Robert Hollingsworth, Nicholas C. Manoukis, Roxana Myers, Paul Bayman, Steven P. Arthurs

**Affiliations:** 1 Coffee Grower & Independent Consultant on Integrated Pest Management, Kailua-Kona, HI 96745, USA; 2 USDA-ARS, PBARC, Hilo, HI 96720, USA; Melissa.Johnson@ars.usda.gov (M.J.); Robert.Hollingsworth@ars.usda.gov (R.H.); nicholas.manoukis@ars.usda.gov (N.C.M.); Roxana.Myers@ars.usda.gov (R.M.); 3 Coffee Grower & Director of Synergistic Hawaii Agriculture Council, Hilo, HI 96720, USA; suzanne@coffeeofkona.com; 4 Department of Biology, University of Puerto Rico-Rio Piedras, San Juan, Puerto Rico 00931, USA; bayman.upr@gmail.com; 5 Department of Entomology, Texas A&M University, College Station, TX 77843-2475, USA; sarthurs@tamu.edu

**Keywords:** *Hypothenemus hampei*, cultural practices, biological control, area-wide monitoring, post-harvest, *Beauveria bassiana*, invasive species

## Abstract

The coffee berry borer (CBB), *Hypothenemus hampei*, is the most significant insect pest of coffee worldwide. Since CBB was detected in Puerto Rico in 2007 and Hawaii in 2010, coffee growers from these islands are facing increased costs, reduced coffee quality, and increased pest management challenges. Here, we outline the CBB situation, and summarize the findings of growers, researchers, and extension professionals working with CBB in Hawaii. Recommendations for the Integrated Pest Management (IPM) program for CBB in Hawaiian Islands and Puerto Rico include: (1) establish a CBB monitoring program, (2) synchronize applications of insecticides with peak flight activity of CBB especially during the early coffee season, (3) conduct efficient strip-picking as soon as possible after harvest and perform pre-harvest sanitation picks in CBB hotspots if needed, (4) establish protocols to prevent the escape of CBB from processing areas and when transporting berries during harvest, and (5) stump prune by blocks. Progress achieved includes the introduction of the mycoinsecticide *Beauveria bassiana* to coffee plantations, the coordination of area-wide CBB surveys, the establishment and augmentation of native beetle predators, and an observed reduction of CBB populations and increased coffee quality where IPM programs were established. However, CBB remains a challenge for coffee growers due to regional variability in CBB pressures, high costs, and labor issues, including a lack of training and awareness of CBB management practices among growers.

## 1. Introduction

Coffee, both *Coffea arabica* and *C. robusta*, is produced on 10 million ha in over 80 countries from tropical and subtropical regions, with over 100 million people employed in this industry [1,2]. In the United States (US), coffee is produced in Hawaii and Puerto Rico, with an estimated combined value of $130 million per year [3]. In Hawaii, coffee is produced in the Kona and Ka’u districts of Hawaii Island on ~2400 ha [4], as well as on ~1400 ha on the islands of Kauai, Oahu, Maui, and Molokai [5]. In Puerto Rico, most of the coffee is produced in the Cordillera Central mountain range in the west–central region, but has recently expanded to the northern coastal plain, occupying a total of about 14,000 ha from ~4500 farms, which is down from 77,000 ha a century ago [6]. Much of the coffee industry in both countries is considered a high value specialty crop [5,7]. In general, production costs are higher in these regions; for example, Hawaiian-grown coffee is five to seven times more expensive compared with the global average [1].

The coffee berry borer (CBB), *Hypothenemus hampei* (Ferrari), is a recent problem for US coffee growers. In Hawaii, CBB was first detected in the Kona District of Hawaii Island in 2010, and has since spread to most of the ~800 coffee farms on the island. More recently, CBB was detected on the neighboring Hawaiian Islands of Oahu (2014) and Maui (2016) [8]. CBB was detected earlier in Puerto Rico (2007), and is now widespread across most coffee-growing regions [9]. While coffee production has historically been profitable, the presence of CBB has altered the landscape. For example, high standards of coffee quality such as ‘Extra fancy’, ‘Fancy’, and ‘Number One’ grades (valued at US$ 30–120/lb. for the processed crop) were drastically affected by CBB during 2011–2013 in Hawaii, which affected the export markets. Production costs have increased 10–15% during this period [10].

## 2. Challenges in Hawaii and Puerto Rico

Adopting IPM programs for CBB while controlling production costs is essential, since this pest may infest up to 100% of berries at harvest if no control actions are taken [11]. IPM programs focusing on pesticides, crop sanitation, and biological control have been developed in other regions for CBB [2,12,13,14]. Many IPM examples emphasize cultural controls, such as conducting frequent harvesting to remove CBB breeding habitats. Although labor intensive, an efficient collection of the mature and older dried berries can remove >80% of the CBB population [15]. Alcohol-baited traps are also widely used to monitor adult CBB populations, so that problem areas within farms may be identified, and insecticide applications can be better guided against the colonizing female CBB [16,17,18]. Biological control strategies include the release of exotic parasitic wasps in coffee plantations [13,19,20].

Due to variability in environmental and cultural practices and specific regulatory requirements, IPM programs must be developed and tested under local conditions. One issue for the development of IPM programs relying on manual labor is the shortage of workers and relatively high labor costs, around US$12–15 p/h in Hawaii and US$7.50–10 p/h in Puerto Rico. A shortage of harvesting labor contributed to recent CBB outbreaks in Puerto Rico, where >20% infestation was observed in 40% of the coffee farms sampled [21]. In Puerto Rico, an additional challenge was created by the devastation of Hurricanes Irma and Maria in September 2017, which destroyed 80% of the commercial coffee plantations and 90% of the infrastructure, according to preliminary estimates [22]. Loss of shade trees means that coffee plants are exposed to more sunlight and higher temperatures. Coffee production will likely be halted for at least one year, to allow surviving plants to recover and replacements to grow. Fallen trees, landslides, broken bridges and power outages present logistical problems for re-establishing the industry. There is also a demographic challenge: most growers are over sixty years and some do not plan to replant. Hurricanes may cause further contraction and consolidation of the industry. In the interim, unharvested berries may lead to a rapid expansion of CBB population across Puerto Rico’s coffee growing region.

Despite these challenges, there is a need to maintain high quality standards of the specialty coffee that is exported from these islands. Below, we review ongoing efforts to develop IPM programs for CBB. We report on our preliminary findings from Hawaii, and provide a perspective for implementing IPM programs on the islands (including Puerto Rico) a decade after this pest invaded the United States.

## 3. Research Progress from Hawaii

Given that CBB is still a relatively newly established pest, its seasonal phenology in Hawaii is not well understood. Additionally, it is not clear how effective current management practices (e.g., insecticides, harvesting, and pruning) are at controlling the CBB populations. Starting in 2016, researchers at the United States Department of Agriculture’s Agricultural Research Service (USDA-ARS) in Hilo, and the University of Hawaii initiated an area-wide CBB monitoring program on 14 Hawaii Island coffee farms. The Synergistic Hawaii Agriculture Council (SHAC) concurrently conducted field surveys on 17 different coffee farms on Hawaii Island.

All of the farms were located in either the Kona (a low humidity region on West Hawaii Island) or Ka’u districts (South Hawaii Island, where growing conditions are ideal) [23]. Most coffee farms were family-owned small-holder enterprises located between 204 and 778 masl, and varied from 0.5 to 1.5 ha. Climate was moderate year-round, i.e., average daily temperatures were 20–25 °C, relative humidity was 70–90%, and average monthly rainfall ~10 cm (measured on farms from May 2016 to February 2017). Most plantings were *C. arabica* var. Kona typica, red bourbon, red caturra, and yellow caturra, with densities ranging from 1500–2800 tree/ha. Harvesting was conducted from August through December (Kona) and from September through February (Ka’u). Many growers sprayed the mycoinsecticide *Beauveria bassiana* (Botanigard ES and Mycotrol O) alone or in combination with other additives (e.g., kaolin clay, pyrethrins, and spreaders/stickers) to manage CBB. Based on management records, growers applied an average of 5.3 (range 4–9) applications per site in the SHAC survey, while in the USDA survey growers applied an average of 6.4 (range 3–12) applications per site.

## 4. CBB Monitoring Surveys

The CBB survey conducted by the USDA included two main components. Firstly, CBB flight activity was monitored at two-week intervals in all of the farms, using alcohol-baited funnel traps (Brocap^®^, CIRAD, Indonesia). Traps were hung from trees (~1.2 m height) following published recommendations [16,18]. Secondly, berry infestation data was collected from eight farms in Kona and six farms in Ka’u following a sampling method developed for Hawaii [24]. In this method, each farm was divided into polygons ~335 m^2^, and a single tree per polygon was randomly selected. From this tree, a representative branch at chest height was sampled for the number of green berries (pea size or larger), infested green berries, green berries with evidence of *B. bassiana* fungus, and raisin berries (over ripe). The number of coffee trees sampled varied from 14–25 per 0.5–1 ha lot. The SHAC survey sampled 30 coffee trees per lot (0.5–1.5 ha). A branch in the middle of each tree was randomly selected, and developing berries were counted and examined for CBB entry holes.

For both surveys, ~3 infested green berries per branch (i.e., ≥30 per lot) were collected and dissected to assess the position of the CBB inside. Infested berries were categorized as AB or CD depending on whether the CBB had penetrated the endosperm (CD position) or remained nearer the surface (AB position). In general, beetles in the CD position are poor targets for *B. bassiana*, since it is more difficult for the fungus to penetrate deep into the berry and establish an infection; moreover, some CBB reproduction may have occurred by this stage [15]. The USDA survey noted the proportion of CBB infected with *B. bassiana* in dissected berries, which was identified by symptomatic white mycelium and spores [25].

Additional information collected during USDA surveys included details on coffee crop phenology, farm weather data, and grower management practices. The aim of these surveys is to better understand the seasonal phenology of CBB in Hawaii, including infestation rates and stages, dispersal periods, and survival between seasons. The long-term goal of the USDA area-wide monitoring program is to use this baseline information to predict pest outbreaks and parameterize models to support treatment recommendations for CBB in Hawaii and Puerto Rico.

### 4.1. Flight Activity Trends

**Figure 1:**
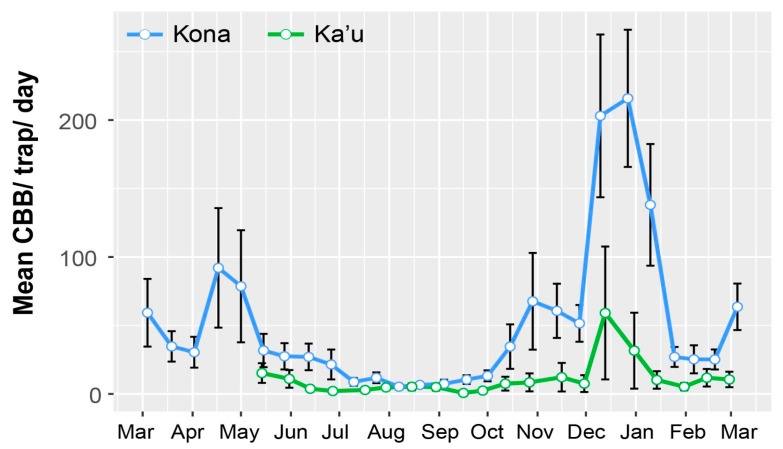
Flight activity of coffee berry borer (CBB) during the 2016–2017 growing season in the Kona and Ka’u districts, Hawaii Island, inferred by the number of CBB caught per day in funnel traps (Brocap^®^) baited with a 3:1 methanol-ethanol attractant. Peak flight activities occurred from March–May and October–December. Data are mean ± SEM from 3–9 traps per farm (n = 14), from the USDA monitoring program.

**Figure 2:**
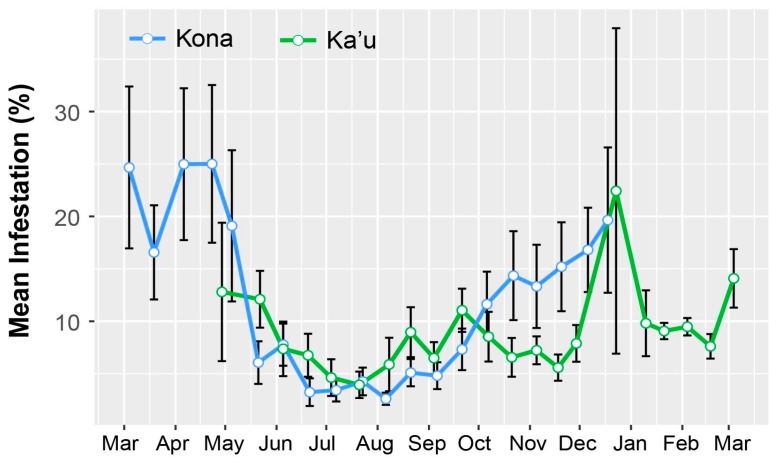
Infestation of green coffee berries by coffee berry borer (CBB) in Ka’u and Kona during the 2016–2017 growing season. Data are mean ± SEM for eight farms in Kona and six farms in Ka’u as part of the USDA monitoring program.

Surveys revealed peak CBB activity occurring around harvest periods in December/early January, with additional flights in March–May and October–November (Figure 1). Flight activity coincided with high berry infestation (Figure 2). Similar results were reported earlier across both districts [26], with the highest trap catches recorded during harvest in December and January. While trap catches were generally higher in Kona, there was substantial variation among farms, as well as within farms.

### 4.2. Berry Infestation Trends

**Figure 3:**
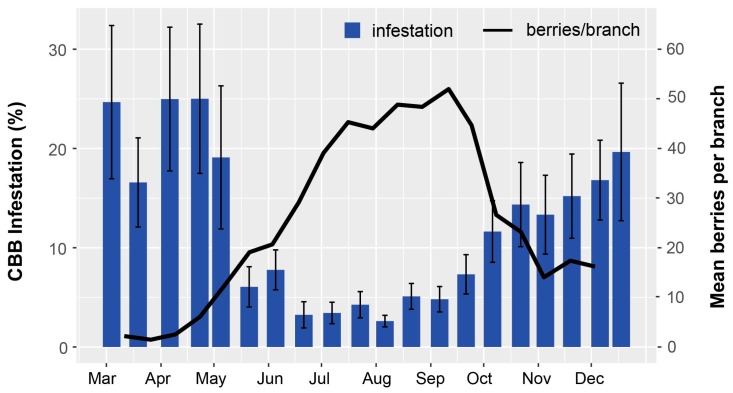
Coffee berry borer (CBB) infestation (mean ± SEM) and coffee plant phenology averaged across eight farms in Kona during the 2016 coffee-growing season. Data were collected during USDA field surveys, and percent infestation was calculated based on the number of green berries that were observed to have a CBB entrance hole out of the total number of green berries counted for each farm/sampling date. The low infestation observed from May–September coincides with high berry production.

**Figure 4:**
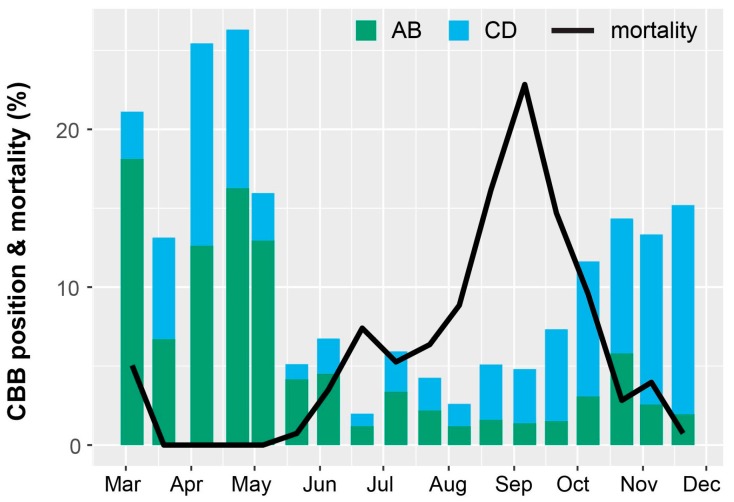
Distribution of female coffee berry borer (CBB) in either AB (endosperm not penetrated) or CD (endosperm penetrated) position in infested green berries, and mortality of the CBB associated with the *B. bassiana* fungus. Estimates of CBB positions and mortality are based on dissections of infested green berries that were collected from each farm/sampling date during the USDA monitoring survey. Proportions of CBB in the AB and CD positions were calculated the 2016 Hawaii IPM guidelines [27]. Data are averaged across eight farms in Kona in 2016.

**Figure 5:**
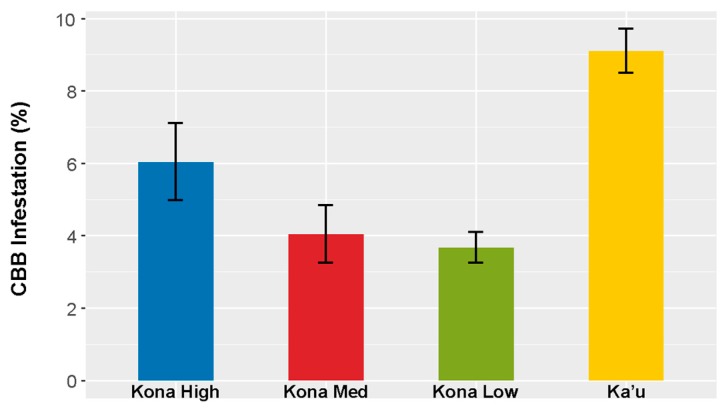
Coffee berry borer (CBB) infestation averaged across the 2016–2017 season in commercial coffee farms from Kona (separated into high (>573 masl), medium (426–434 masl), and low (<313 masl) elevations) and Ka’u districts on Hawaii Island. Data are mean ± SEM from 17 farms monitored between May 2016 and January 2017 in the Synergistic Hawaii Agriculture Council (SHAC) survey.

**Figure 6:**
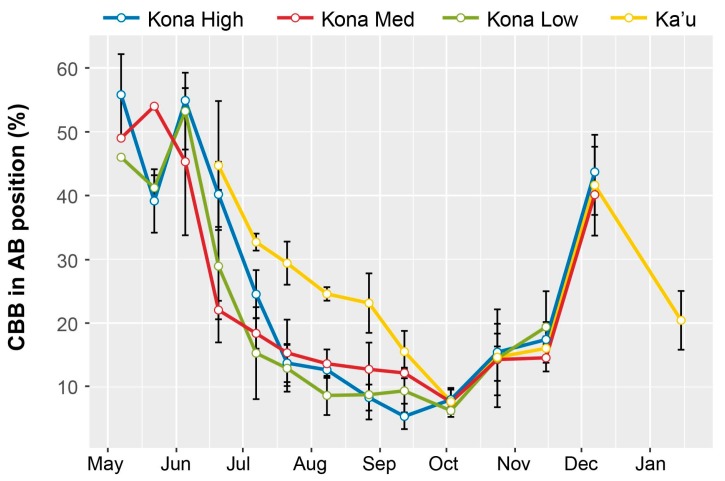
Estimates of coffee berry borer (CBB) in the AB position on commercial coffee farms in Kona (separated into high (>573 masl), medium (426–434 masl), and low (<313 masl) elevation farms) and Ka’u districts on Hawaii Island. Data are means and SEM from 17 farms monitored during the SHAC survey between May 2016 and January 2017 (end of harvest season).

**Figure 7:**
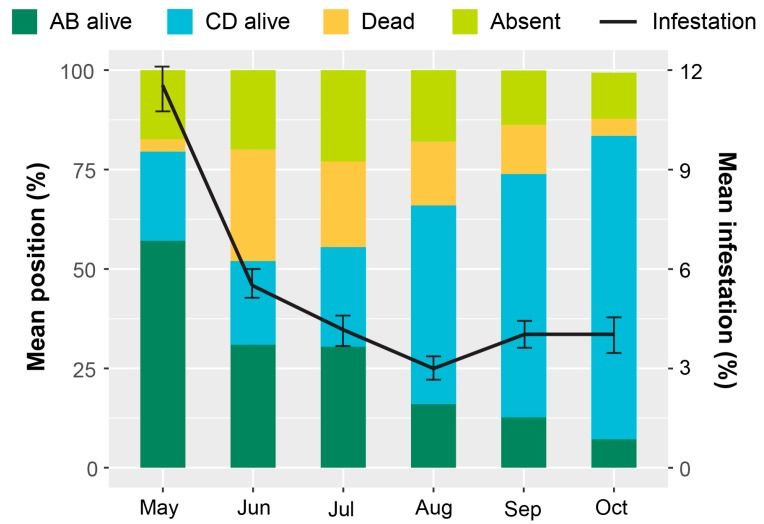
Mean CBB infestation (±SEM), positions (AB/CD alive), absence (hole bored but CBB missing from berry), and mortality. Evaluations were conducted on Kona coffee farms in 2017 during the SHAC survey on 10 (May), 26 (June), 41 (July), 21 (August), 17 (September), and 16 (October) coffee farms, respectively.

The highest periods of berry infestation occurred from March to May, and again in late October through the end of harvesting in December, when on average ~20% of berries were infested (Figure 3). The increased infestation at harvest likely came from second or third generation CBB colonizing new berries. Despite the surplus of berries from June to September (Figure 4), infestation rates were low during this time (Figure 3), likely due to minimal flight activity (Figure 1). The USDA survey also revealed that early berry development (March–May) is when most CBB are in the AB position (Figure 4). This represents a key period for *B. bassiana* to be applied, since later in the season, most CBB were in the CD position where they are poor targets for the fungus [15]. Although the highest mortality caused by *B. bassiana* occurred in September–October (Figure 4), the mean mortality by *B. bassiana* across the entire 2016–2017 season in Ka’u was twice that observed in Kona (12% vs. 6%, respectively). This may reflect more favorable weather conditions (i.e., higher humidity) for the fungus in Ka’u. The SHAC survey noted highest berry infestation rates occurring on farms at high altitudes (>573 masl), and lowest infestation rates at lower altitudes (<313 masl; Figure 5). Temperature, relative humidity, and rainfall vary significantly with elevation, and likely play an important role in CBB population dynamics across Hawaii Island. While the SHAC survey noted berry infestation rates were higher in Ka’u compared with Kona sites (Figure 5), the opposite trend was seen in the USDA survey (Figure 2), and this likely reflects differences in management practices among farms rather than true differences between districts. A similar distribution for the percentage of CBB colonizing coffee berries was noted between districts, and among elevations within the Kona district, with major peaks occurring from May–June, and again in December at harvest (Figure 6). Lastly, SHAC surveys conducted in Kona in 2017 showed that most CBB were in the AB position early in the season and transitioned to the CD position by June/July, while peak mortality and abandonment of berries (i.e., absence) occurred in June (Figure 7). Economic injury levels based on infestation levels, CBB positions, as well as trap catch data are being investigated.

## 5. Cultural Control Methods

Cultural control practices conducted in other coffee-growing regions of the world [14,20,28,29] are more challenging in Hawaii and Puerto Rico given the current labor issues. These practices include the periodic collection of ripe and over-ripe (raisin) berries before and during the harvest season and post-harvest sanitation. In Hawaii, growers are generally advised to remove all remaining berries (unripe, ripe, and over-ripe) from the trees after harvest in a process called ‘strip-picking’ [23]. This practice, if done well, removes all CBB habitats (except those provided by berries on the ground and feral coffee), and reduces the number of CBB re-infesting the following year’s coffee crop. Collection of fallen berries from the ground is a cultural practice recommended in some other regions, including Central and South America, to reduce CBB populations [20,28]. However, this practice is not conducted in Hawaii and Puerto Rico, in part because the cost of labor is prohibitive.

### 5.1. Harvesting Efficiency

The SHAC survey evaluated harvesting efficiency among 11 coffee farms from the Kona District in 2016. The objective was to quantify how harvest practices influenced the residual populations of CBB, according to previously determined criteria [15]. At low to moderate CBB densities (<5% infestation), the manual removal of berries is rated as ‘excellent’ when ≤5 mature berries per coffee tree remain after harvest, ‘good’ when 6–10 berries remain, and ‘bad’ when >10 berries are left on coffee trees. A total of 36 harvesting rounds were evaluated in different coffee lots between 0.5 and 1.5 ha. After each harvest round by workers, 15 coffee trees were randomly selected per lot, and all ripe and over-ripe berries were collected and counted.

**Figure 8:**
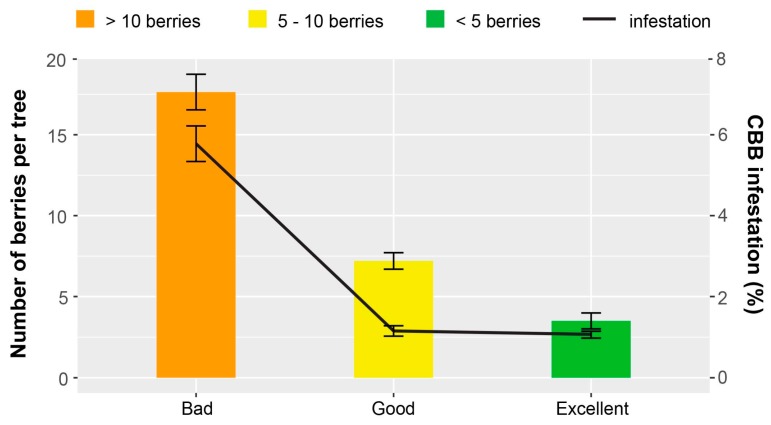
Efficacy of harvesting practices (SHAC survey) on 11 commercial coffee farms from the Kona district of Hawaii Island during the 2016 harvest. Harvesting practices are evaluated based on the scale of Bustillo et al. (1998); <5 berries/tree is rated ‘excellent’, 6–10 berries/tree is ‘good’, and >10 berries/tree is ‘bad’. Data are mean ± SEM from 36 harvesting rounds. Overall, 69.7% of the harvesting rounds were rated as bad, 21.2% were rated as good, and only 9.1% were rated as excellent.

An average of 13.9 berries/tree (range 2.5–31.4) remained post-harvest in 2016. Overall, only one third (30.3%) of harvesting rounds by coffee pickers were classified as ‘good’ or ‘excellent’ (i.e., >10 berries/tree), with the majority rated as ‘bad’ (Figure 8). In 2015, an average of 20–45 berries left per tree after harvesting was reported [30]. Low CBB infestation levels (<2%) correspond with ‘excellent’ and ‘good’ harvesting practices; in contrast, ‘bad’ harvesting practices correspond with higher CBB infestation (>5%). This provides circumstantial evidence that workers are becoming more efficient, possibly as a result of ongoing training workshops provided by the SHAC program and the Extension Service at the University of Hawaii. However, this is not conclusive, since there may have been variability in training practices or experience, and different workers may have conducted the harvesting in 2015 and 2016. Mechanical harvesters are employed in some regions, and while they reduce labor costs, they are not considered as efficient, and may leave 12–20% of berries uncollected [31].

Additional factors, including management practices and environmental variables, may also influence the harvesting efficiency between years. For example, the harvest season is 3–4 months (September–December) long in Kona, and 4–8 months (September–April) long in Ka’u. In contrast, the coffee harvest in Colombia is divided into a major harvest (September–December) and minor harvest (March–May) [15]. These differences in harvest seasons are linked to weather patterns, which affect the phenology of coffee plants and insect populations. Since berries are available year-round in Columbia and for most of the year in Ka’u, CBB control in these regions are more challenging. However, observations from Colombia also show that efficient harvest practices can still be used to manage this pest since low-cost labor is available [29]. More detailed studies are needed in Hawaii to determine how management and weather variables affect harvesting efficiency and subsequent CBB populations.

### 5.2. Strip-Picking

The post-harvest stripping of all berries (green, ripe, and over-ripe) may reduce CBB survival between seasons [23]. In coffee plantations with only a single flowering period (3–4 months) and a relatively short harvesting season, a single strip-pick may sufficiently reduce CBB. However, since strip-picking practices tend to be variable among farms [31], we conducted a preliminary survey on the importance of the timing of strip-picking with respect to the final harvest. Three different farms in the same region of Kona growing *C. arabica* var. *typica* were selected; each farm had similar numbers of verticals per tree (3–4), were ≤3 years old, and had CBB activity monitored by traps. In two farms, strip-picking was conducted during early December close to harvesting, but was delayed by two months in the third farm.

**Figure 9:**
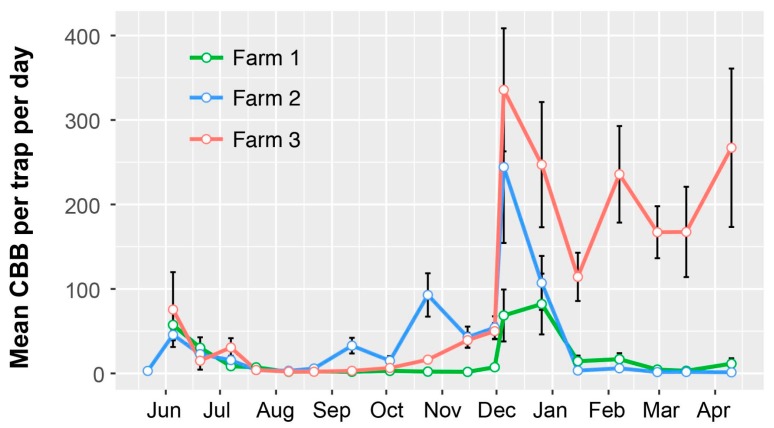
Number of coffee berry borer (CBB) captured per day in alcohol-baited funnel traps on three coffee farms in Kona (0.5–1 ha and 1750–2000 trees/ha, 280–300 masl) where strip-picking was conducted. In farms 1 and 2, strip-picking was conducted during early December 2016, and in farm 3, during late January 2017. Data are mean ± SEM for each farm with 5 traps/farm.

Results showed that CBB initially increased in all of the farms during the harvest period in December. However, there was a fairly rapid decline in CBB activity in both farms where strip-picking was conducted soon after harvest, compared with the farm where strip-picking was delayed for nearly two months (Figure 9). Moreover, in the latter farm, relatively high CBB activity (i.e., >100 CBB/trap/day) was observed during the survey, which ended in April. This observation supports the idea that early strip-picking of all berries (close to the end of harvest) will reduce the residual CBB population available to impact the following crop. However, it is also important to remove and destroy berries collected during strip-picking.

### 5.3. Pruning by Block

Periodic pruning at the end of the season is a standard practice to maintain the size and vigor of larger coffee trees [23]. Regularly pruned trees are also easier to harvest and spray by workers [31]. Pruning can also potentially remove CBB habitat (such as bark chinks), and thus provides an additional cultural control method. In some regions, coffee trees are periodically pruned in entire blocks, a practice which significantly reduced the number of CBB in that area [12,13,14,15]. However, in Hawaii, traditional ‘Kona Style’ and ‘Beaumont–Fukunaga’ pruning practices mix age classes and pruning within blocks [23]. Under this system, recently pruned trees can be more easily re-infested by CBB from adjacent trees.

**Figure 10:**
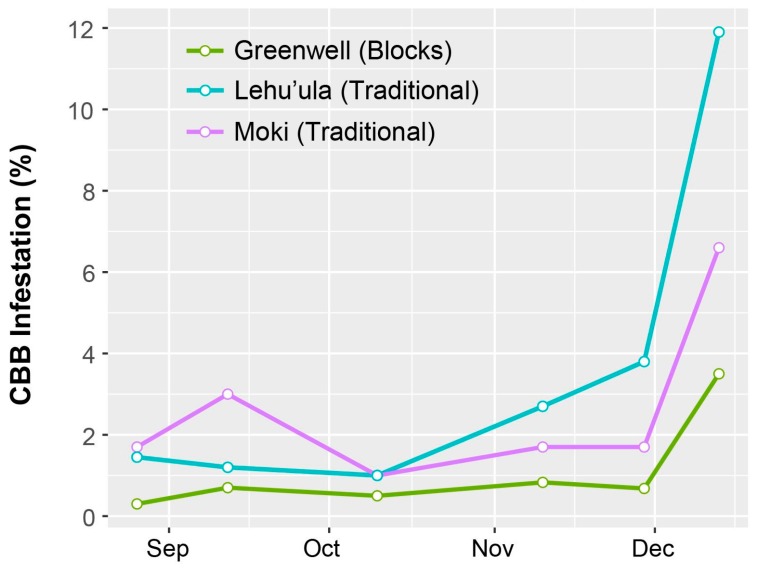
Proportion of berries infested with coffee berry borer (CBB) at harvest in coffee lots receiving prior ‘block’ or traditional ‘Beaumont–Fukunaga’ pruning in Kona, Hawaii Island. Data are based on 30 randomly selected trees/lot in ~1 ha coffee farms located in Kealakekua, Kona at 500 masl (Greenwell Farms) and 400 masl (Lehu’ula Farms), and Captain Cook, Kona at 600 masl (Moki Farms).

We investigated the value of block pruning in a 1.5 ha coffee farm (Greenwell) located in Kona, Hawaii Island. Following block pruning in February and March 2015, CBB populations were tracked in this farm the following (2016) season. Two adjacent farms receiving traditional pruning by ‘Beaumont–Fukunaga system’ were selected for comparison. All of the lots were planted with *C. arabica* var. *typica*. Results showed that CBB infestation levels in these pruned lots increased at harvest in all farms, but remained lowest in the farm receiving block pruning, which provides support for this approach (Figure 10). Total defects in the processed dried coffee (10 lb sample collected post-harvest), including damage caused by CBB, was also lower in the block-pruned farm (3.9%) when compared with the traditional-pruned farms (Moki = 10.2% and Lehu’ula = 10.6%).

Stump pruning by block after strip-picking is currently recommended by the University of Hawaii to minimize CBB habitat [27]. However, it may be necessary to monitor blocks after pruning and apply insecticides as needed to prevent CBB populations from spreading to other areas [32]. Alcohol traps may be installed inside pruned coffee lots to determine when CBB adults emerge.

### 5.4. Post-Harvest

Controlling the CBB in processing facilities is needed to stop emerging beetles from re-infesting adjacent coffee lots [27,30]. Strategies to prevent the escape of CBB post-harvest in other regions include tying burlap bags used for berry collection, screening the wet mill, silos, and pulp pits with transparent plastic smeared with grease, and using a mechanical dryer for processed coffee [14,15]. In addition, the use of alcohol-baited traps may capture CBB adults that emerge from processed coffee in the wet and dry mill. Although these practices are recommended in Hawaii [27], we observed that many growers have yet to adopt them. A similar situation was observed in coffee farms in Puerto Rico [31]. Since the escape of CBB from coffee processing facilities (wet and dry mill) is a source of CBB, the feasibility of control strategies is currently being evaluated in Hawaii. Recent research highlights the potential of freezing as a CBB treatment used in shipping for green coffee [33].

## 6. Biological Control

Several types of biological control, including classical, inundative, and conservation, have been explored for the control of the CBB worldwide [2,12,13,20]. Ongoing work in Hawaii is currently exploring several approaches with biological control agents to help control and mitigate the effects of the CBB, and reduce the need for labor-intensive cultural control strategies.

### 6.1. Beauveria bassiana

Products based on the entomopathogenic fungus *Beauveria bassiana* are used against CBB in several regions [2,13,34]. This fungus, which is sold as Botanigard^®^ and Mycotrol^®^, is among the few insecticides registered for Hawaiian coffee, having been approved for use by the Hawaii Department of Agriculture (HDOA) in 2011. When it was registered, there was an effort to persuade farmers to monitor and spray Botanigard^®^ if they had the CBB. The SHAC obtained a Federal grant to subsidize the cost of *B. bassiana* to growers, which resulted in almost 80% of the acreage of planted coffee on Hawaii Island and Oahu (about 1900 ha) being treated in the subsequent four years. On coffee farms that spray *B. bassiana*, it is common to see CBB-infested fruits with a white, powdery spot near the entry hole. The white spot is a sporulating colony of *B. bassiana*, which attacks and kills CBB within fruits. The SHAC survey observed CBB mortality rates up to 28% due to *B. bassiana* applications in Kona during 2017 (Figure 7), and similar results were observed in the USDA survey (Figure 4).

Several insecticide applications per year are recommended at times of peak CBB flight activity. During the SHAC surveys, farmers applied *B. bassiana,* either alone or combined with kaolin clay (Surround^®^WP), an average of 5.3 times in the 2016/17 coffee production cycle (data for Kona). Most applications were made early season, when >25% of the CBB in developing green berries were in the AB position (based on results described above). Application costs (four seasonal applications) were estimated at US$240/acre for Hawaii [35]. In Puerto Rico, fewer coffee farmers (about 20%) applied *B. bassiana* (sold as Mycotrol^®^) at least once a year, according to a recent study [21].

There may be potential to develop improved mycoinsecticides in Hawaii and Puerto Rico. Endemic *B. bassiana* strains are genetically diverse in Hawaii, and presumably adapted to local conditions. However, these endemic strains are not in the ‘clade A’ of *B. bassiana* strains, which is a genetic group to which the common ‘GHA’ strain used in Botanigard and Mycotrol belongs [25]. Strains endemic to Puerto Rico were as virulent to CBB as the GHA strain, but more persistent in the environment [36]. However, the small market size may dissuade commercial investment and the registration of new *B. bassiana* products.

### 6.2. Predators

Exotic parasitoids have been released against CBB in several countries [2,12,13]. Releases have not been approved in Hawaii, due in part to concerns about native bark beetles. However, endemic predators such as the flat bark beetle *Cathartus quadricollis* (Coleoptera Silvanidae) and *Leptophloeus* spp. (Coleoptera: Laemophloeidae) are common in macadamia crops, and can be effective predators of CBB, especially in fallen berries [37,38]. These native predators could help regulate CBB outbreaks. Ongoing research is currently exploring conservation and augmentation strategies for *C. quadricollis* through the provision of on-farm ‘breeding stations’ [38] as well as the introduction of the CBB parasitoids *Phymastichus coffea* (Hymenoptera: Eulophidae), *Cephalonomia stephanoderis,* and *Prorops nasuta* (Hymenoptera: Bethylidae) [30].

### 6.3. Entomopathogenic Nematodes

**Figure 11:**
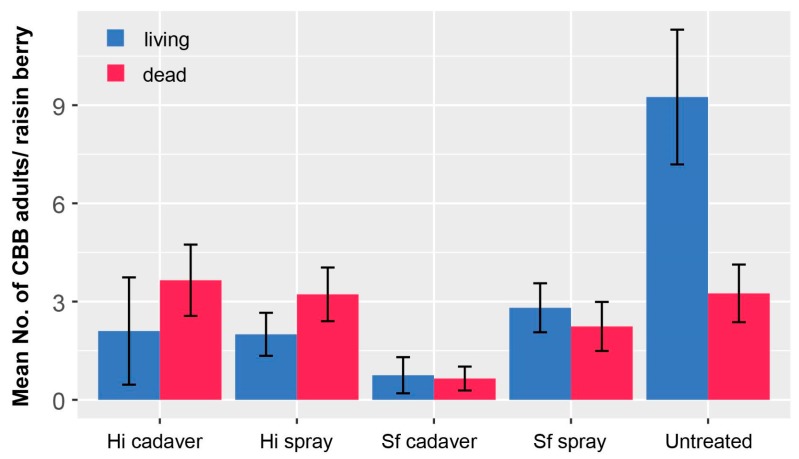
Field study evaluating the effect of two endemic nematodes on coffee berry borer (CBB) survival in raisin berries on the ground in Hilo, HI. Hi = *Heterorhabiditis indica* strain OM-160; Sf = *Steinernema feltiae* strain MG-14; spray = 1500 infective juveniles/ml; 14 ml/tree; cadaver (i.e., two nematode-infected mealworm cadavers/tree). Data are a randomized complete design with five replicates.

Entomopathogenic nematodes in the genera *Heterorhabditis* and *Steinernema* have been explored for the control of CBB in green and raisin berries on the ground in Hawaii Island. One study reported that aqueous sprays of *S. carpocapsae* resulted in mortality of 17.1% of CBB larvae and 4.7% of CBB adults [39]. Currently only *S. carpocapsae* (Weiser) is permitted to be imported to Hawaii. However, *S. feltiae and Heterorhabditis indica* are already present in Hawaii, and can be reared for field tests without restriction. Preliminary field tests with two nematodes endemic to Hawaii (*S. feltiae* strain MG-14 and *H. indica* strain OM-160) were conducted by spraying CBB-infested fallen berries with infective juveniles, or placing mealworm (*Tenebrio molitor*) cadavers previously infected with nematodes among the infested berries. Fifty trees were evaluated with 10 berries per tree. Dissection of the berries two weeks after inoculation suggested that the nematodes did not cause high mortality, but resulted in abandonment of the berry (Figure 11). This abandonment behavior in response to nematodes was observed in laboratory bioassays [35]. Post-harvest treatment with nematodes resulting in direct mortality or berry abandonment when few berries are present on trees may reduce the number of surviving CBB available to infest the following year’s crop if followed by an insecticide or *B. bassiana* spray as part of an IPM program.

## 7. Recommendations and Lessons Learned

Compared with other regions, little research has been conducted on CBB in the United States, which likely reflects the smaller market and relatively recent arrival of this pest. However, IPM approaches developed in other regions can help US growers cope with this pest. These approaches will continue to be tailored to Hawaii and Puerto Rico’s unique environmental and socioeconomic conditions, updated as new information becomes available, and recommended to growers via outreach done by the University of Hawaii Cooperative Extension Service and USDA [27].

**Figure 12:**
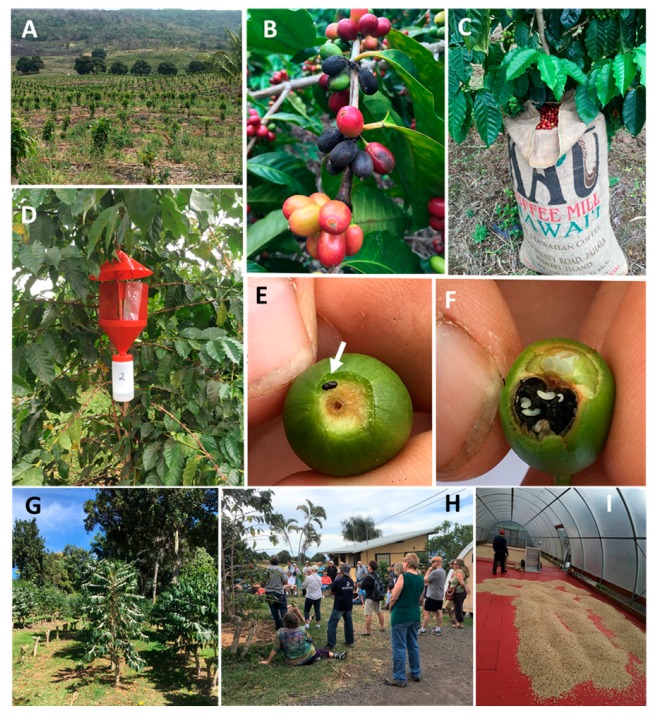
Coffee plantation in Kona, HI pruned by blocks (**A**), ripe, over-ripe, and raisin berries are collected during cultural sanitation (**B**), open burlap bag allows the escape of CBB (**C**), red alcohol-baited funnel trap for CBB (**D**), CBB in the AB position (**E**), CBB in the CD position with progeny (**F**), traditional ‘Beaumont–Fukunaga’ pruning (**G**), training coffee growers (**H**), enclosed dry mill prevents escape of the CBB (**I**). Photos by Luis F. Aristizábal.

**Figure 13:**
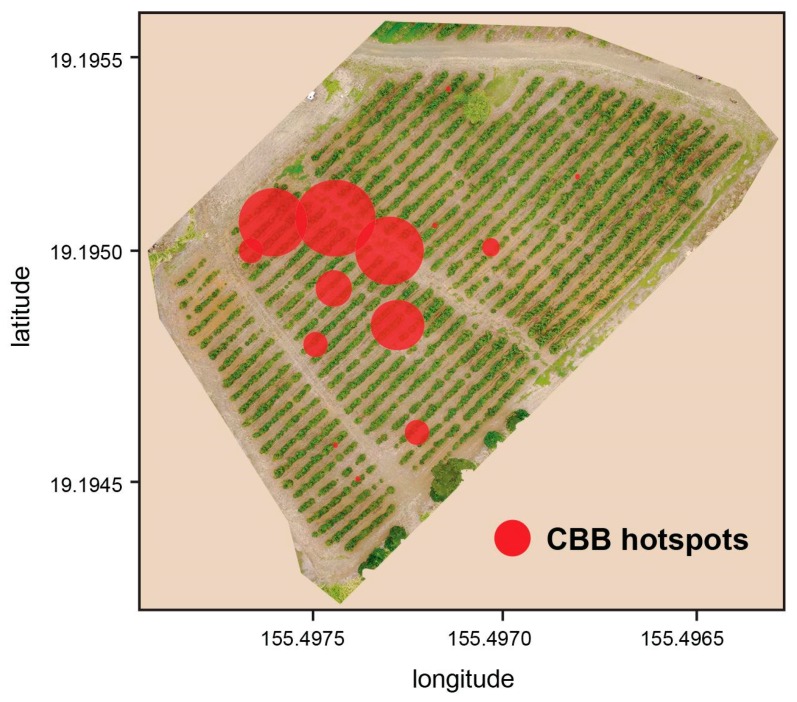
Map of a Ka’u coffee field showing coffee berry borer (CBB) hotspots observed during a USDA monitoring survey in June 2016. The size of the red circle is proportional to the number of green infested berries on a sampled branch.

Currently, post-harvest strip-picking and stump pruning by block are key practices that limit CBB survival between seasons (Figure 12A,B,G). Area-wide monitoring of CBB provides a framework for an IPM program that is customized to Hawaii and Puerto Rico’s heterogenous landscapes. The ‘30-tree sampling plan’ [15] helps growers to identify ‘hot spots’ relatively easily (Figure 13) where sanitation and/or insecticide treatments are warranted. Data on berry infestation rates and stages can be used to determine appropriate times to spray *B. bassiana* based on IPM guidelines [27]. For example, application of *B. bassiana* is most effective when synchronized with CBB in the AB position (Figure 12E,F), which is observed during April through July (Kona) and in May through August (Ka’u), and during harvest in November through February. Methods to contain and kill CBB during and post-harvest and when transporting berries, are also recommended (Figure 12C,I). Trapping data from different regions will provide information on effective management practices for CBB in Hawaii (Figure 12D). Farmers can make their own traps from a plastic soda bottle to save money [40]. Additional efforts are exploring the development of trap action thresholds that growers can use [26]. For biological control, the flat bark beetle project managed by the University of Hawaii Cooperative Extension Service is teaching growers about these native CBB predators and how to augment populations on their farms [41].

In an attempt to prevent the spread of CBB to other islands, the HDOA has placed a quarantine on regulated coffee items (unroasted coffee, coffee plants and plant parts, used coffee bags, and coffee-harvesting equipment) shipped from Hawaii Island and Oahu to all other islands. Inspection by state officials and treatments such as fumigation, freezing, or heating and bagging, is also needed prior to shipping regulated items (plants, green beans, and used equipment, etc.). Such items must have a valid permit issued by the APHIS Plant Quarantine Branch before transport is allowed [33,42].

We have learned several lessons regarding CBB management in Hawaii. For example, while alcohol-baited traps are an effective monitoring tool [18], native non-target beetles in the same genus, such as *H. obscurus*, are also captured in traps and can be numerous (10–20% of the annual mean catch in Kona and Ka’u, respectively; data from USDA surveys). Thus, sorting and identifying specimens, as well as investigating relationships between trap catches and infestation rates in coffee berries may be needed. Coffee growers and field workers may need training to accomplish optimal harvesting practices (Figure 12H). As noted above, labor issues limit cultural control practices. The variable climate and topography also influence CBB phenology in Hawaii [18,26], Puerto Rico [43], and elsewhere [44]. These differences make area-wide recommendations problematic. The use of gibberellic acid to synchronize flowering and condense harvesting periods may be useful [45].

## 8. Conclusions

Research worldwide confirms that no single strategy will control CBB. Currently not all growers adopt IPM strategies in Hawaii and Puerto Rico. However, the situation may change if CBB continues to spread and more growers are impacted. Although a number of strategies, including sanitation and *B. bassiana* sprays, are now recommended to growers by the Cooperative Extension at University of Hawaii and USDA-ARS, it will take time for growers to adopt new practices.
